# Understanding Climate Feedbacks and Sensitivity Using Observations of Earth’s Energy Budget

**DOI:** 10.1007/s40641-016-0047-5

**Published:** 2016-09-01

**Authors:** Norman G. Loeb, Wenying Su, Seiji Kato

**Affiliations:** NASA Langley Research Center, Hampton, VA USA

**Keywords:** Climate feedback, Climate sensitivity, Satellite, Earth radiation budget, Observational constraint

## Abstract

While climate models and observations generally agree that climate feedbacks collectively amplify the surface temperature response to radiative forcing, the strength of the feedback estimates varies greatly, resulting in appreciable uncertainty in equilibrium climate sensitivity. Because climate feedbacks respond differently to different spatial variations in temperature, short-term observational records have thus far only provided a weak constraint for climate feedbacks operating under global warming. Further complicating matters is the likelihood of considerable time variation in the effective global climate feedback parameter under transient warming. There is a need to continue to revisit the underlying assumptions used in the traditional forcing-feedback framework, with an emphasis on how climate models and observations can best be utilized to reduce the uncertainties. Model simulations can also guide observational requirements and provide insight on how the observational record can most effectively be analyzed in order to make progress in this critical area of climate research.

## Introduction

Climate is determined by the amount and distribution of incoming solar radiation absorbed by Earth. In response to energy imbalances, complex processes give rise to energy flows within the atmosphere, hydrosphere, lithosphere, cryosphere, and biosphere occurring over a range of time-space scales. For an Earth system in equilibrium, these energy flows must produce outgoing longwave radiation at the top of atmosphere (TOA) that is equal to the incoming absorbed solar radiation. The coupled nature of the system is such that external perturbations to the Earth’s energy budget impact all of the Earth subsystems to varying degrees. The defining challenge for climate science is to understand and predict the timing and intensity of the changes for a range of space-time scales in response to natural phenomena and man’s activities.

The Earth’s surface temperature is expected to rise between 1.5 and 4.5 °C in response to a doubling of atmospheric CO_2_ concentrations [1]. Despite much effort by the climate science community, the large range of uncertainty has not narrowed appreciably over the past 30 years. A key reason is due to the representation of climate feedbacks in climate models. Increased CO_2_ in the atmosphere alters the Earth’s energy balance by reducing how much thermal infrared radiation is emitted to space. Most of the excess energy into the system initially ends up being stored in the ocean, but some also heats the atmosphere and land and melts snow and sea ice. To restore a balance between absorbed solar radiation and outgoing longwave radiation, the Earth system must emit more infrared radiation to space. In the absence of other changes, a CO_2_ doubling would require Earth’s temperature to eventually increase ∼1.2 K [2]. However, temperature changes can also alter other processes and properties of the climate system, which can lead to further changes in Earth’s energy balance that can further modify temperature. In addition to an increase in surface temperature, feedbacks associated with water vapor, clouds, snow and ice, and the vertical temperature structure of the atmosphere are important. A climate feedback is quantified through its climate feedback parameter, given by the change in downward TOA flux for a given temperature change. Thus, an increase in downward TOA flux with warming temperatures yields a positive climate feedback parameter.

Climate models agree that feedbacks collectively amplify the surface temperature response to external forcing, but the strength of the feedbacks varies greatly [3]. Water vapor provides the largest positive feedback, and vertical changes in water vapor and temperature are tightly coupled. Accordingly, the sum of the lapse rate and water vapor feedbacks are well represented by the majority of climate models. Feedbacks due to clouds and surface albedo (associated with snow and ice changes) are also positive in all models, but cloud feedbacks are the largest source of uncertainty in current predictions of climate sensitivity. The main stabilizing (negative) feedback comes from the temperature response (Planck feedback), which is well represented in models [3].

Given the large intermodel spread in climate sensitivity due to uncertainties in climate feedbacks, it is reasonable to turn to observations to help narrow the uncertainty. Recent progress on using observations to help constrain individual feedbacks (e.g., water vapor and high clouds) is summarized in Boucher et al. [4]. There is also ongoing work that relies on data from past climate states to estimate climate sensitivity [5]. Here, we focus on studies that use Earth Radiation Budget (ERB) satellite observations for constraining climate feedbacks operating under global warming. We briefly summarize some of the key observational findings to date and discuss the challenges associated with interpreting the results. We also examine the state of current ERB satellite observations and explore issues related to data analysis.

### Recent Estimates of Climate Feedbacks from Satellite Measurements

The use of short-term satellite records to infer climate feedback has been the subject of considerable debate. Given estimates of radiative forcing, it is possible to use observations of the covariability between surface temperature and TOA radiation to infer empirical estimates of climate feedback. Following Gregory et al. [6, 7], Forster and Gregory [8] use a linearized version of the Earth’s global energy balance in which TOA net downward radiative flux is equated with the difference between TOA radiative forcing and the surface temperature change multiplied by the climate feedback parameter. Since the Earth is not in radiative equilibrium, the climate feedback derived under transient warming is often referred to as effective global climate feedback in order to distinguish it from equilibrium global climate feedback [9]. Using data from the Earth Radiation Budget Experiment (ERBE), Forster and Gregory [8] estimate via linear regression a climate feedback parameter of −2.3 ± 0.7 W m^−2^ K^−1^ (1σ uncertainty). Murphy et al. [10] used observations from both ERBE and the Clouds and the Earth’s Radiant Energy System (CERES) to infer a climate feedback parameter of −1.25 ± 0.5 W m^−2^ K^−1^. More recently, Donohoe et al. [11] and Trenberth et al. [12] obtained climate feedback parameters of −1.2 ± 0.5 and −1.13 ± 0.5 W m^−2^ K^−1^, respectively. By comparison, the climate feedback parameter for a system in which only the temperature or “Planck” feedback is operating is −3.2 W m^−2^ K^−1^. Thus, feedbacks other than the Planck feedback (e.g., water vapor, clouds, snow and ice, and the vertical temperature structure of the atmosphere) are collectively positive and therefore amplify the warming. An alternate approach to estimate climate feedback and equilibrium climate sensitivity is to use longer records of upper ocean heat content (OHC) change, forcing, and temperature. These methods as well as others that use satellite TOA radiation data are discussed in more detail in Forster [13].

Forster and Gregory [8] assume that changes in net TOA radiation due to internal variations of the system unrelated to surface temperature are negligible. Spencer and Braswell [14–16] and Lindzen and Choi [17, 18] argue that climate feedback determined by linear regression of short satellite TOA radiation and temperature records is overestimated due to internal variations (e.g., natural cloud fluctuations or weather noise) that can alter surface temperature directly and thereby act as a source of “nonradiative forcing.” Clouds are typically viewed as a climate feedback since in response to surface warming associated with a forcing, they either amplify (positive cloud feedback) or offset (negative cloud feedback) the initial forcing [19]. Based upon a simple linear box model of Earth, Spencer and Braswell [15, 16] claim that atmospheric feedback diagnosis of the climate system remains an unsolved problem due primarily to the inability to distinguish between radiative forcing and radiative feedback in satellite radiative budget observations.

Several studies [19–21] have pointed out significant weaknesses in the Spencer and Braswell [14–16] and Lindzen and Choi [17, 18] analyses. Murphy and Forster [20] and Dessler [19] repeated their analysis and showed that when a more realistic ocean mixed layer depth is used, the correct standard deviation in outgoing radiation is used, the model temperature variability is computed over the same time interval as the observations, and the difference between the linear regression slope and feedback parameter is an order of magnitude smaller than in Spencer and Braswell [14–16]. Thus, temperature variations at short time scales are primarily directly driven by ocean-atmosphere heat exchange, not from cloud fluctuations. The ocean-atmosphere heat exchange is largely controlled by El Niño–Southern Oscillation (ENSO) events, during which the atmosphere gains/losses energy through variations in surface evaporation and precipitation latent heating [22]. During a La Niña event, the ocean column takes up energy, resulting in cooler surface temperatures, and energy is released to the atmosphere during El Nino events, resulting in warmer surface temperatures. Clouds respond to these ENSO-driven forcings at interannual time scales, but many cloud variations on monthly time scales are also a result of internal atmospheric variability, such as the Madden-Julian oscillation. This “weather noise” complicates the linear regression approach, adding uncertainty to regression slopes derived from TOA radiation and surface temperature.

These studies have also highlighted the importance of using a global domain when estimating a climate feedback parameter. Murphy [23] showed that derivation of a climate sensitivity with a linear regression between satellite TOA radiation and surface temperature records for a limited region such as the tropics (as is done in Lindzen and Choi [17]) is ill-posed since heat transport between regions must also be considered. Accordingly, Brown et al. [24] showed that large-scale atmospheric circulation changes are the main reason why positive unforced regional surface temperature anomalies in the tropics and midlatitudes are associated with positive anomalies in regional net downward TOA radiative flux, whereas positive global mean surface temperature anomalies are associated with negative anomalies in global TOA radiative flux.

### Interpreting Short-Term Climate Feedback

In spite of many problems with aspects of some of the earlier studies, it remains an open question whether or not short-term climate feedback (or equivalently, effective climate feedback) derived from observations provides any insight on climate feedback operating under global warming (equilibrium climate feedback). Trenberth et al. [22] question the interpretation of ENSO as an analogue for exploring the forced response of the climate system. Using 10 years of CERES TOA radiation and surface temperature measurements, Dessler [25] and Dessler [26] applied the methodology of Soden et al. [27] and Shell et al. [28] to show that climate feedbacks from short-term observations and global climate model control runs are generally consistent, although there are notable differences in the regional pattern of cloud feedback. They also note that feedbacks for control and forced climate model runs are only weakly correlated, implying that short satellite records likely only provide a weak constraint on climate sensitivity. More recently, Zhou et al. [29] used Coupled Model Intercomparison Project phase 5 simulations to show that cloud feedbacks in response to interannual and long-term surface warming are well correlated owing to a similar low cloud cover decrease with sea surface temperature occurring at both time scales. However, because different forcings produce different patterns of warming [9, 30, 31], and many factors that influence the global radiation budget are sensitive to spatial variations in temperature, the average long-term cloud feedback differs from that at interannual time scales [29]. This, together with the large uncertainty in model representation of low cloud feedback [32], points to the need for further research on how short-term observations can best constrain climate feedback operating under global warming.

Coupled atmosphere-ocean simulations show considerable time variation in the effective global climate feedback parameter under transient warming [7, 13, 31, 33–41]. This implies that the apparent climate sensitivity inferred from observations of effective global climate feedback for different periods will differ from one another even for perfect observations. Armour et al. [9] argue that global climate feedback is linked to the time evolution of regional climate feedbacks, which depends upon the time variation in the geographic pattern of surface warming resulting from the different response times of land, ocean, and sea ice. On decadal time scales, warming of the low-latitude oceans causes strongly negative regional feedback, leading to an effective climate sensitivity that is lower than the equilibrium climate sensitivity. The climate evolution over the next few decades will thus likely depend strongly upon the geographic variations in ocean dynamics, heat uptake, and transport. Other studies explain the time variation in effective climate feedback through a nonlinear relationship between global cloud radiative forcing (CRF) and global surface temperature [34, 42].

Recently, Sherwood et al. [43] reviewed additional concerns relevant to constraining climate feedback with observations. In the traditional framework, feedbacks are associated with processes and properties that respond to surface temperature changes. The feedbacks alter Earth’s energy balance by enhancing or offsetting the initial forcing. Notable examples of feedbacks tied to temperature are the water vapor and snow-ice albedo feedback. However, recent modeling studies [44–48] have pointed out that the forcing itself can lead to changes that can alter Earth’s energy balance independent of surface temperature. For example, increased CO_2_ concentrations can alter the vertical temperature lapse rate in the middle and lower troposphere by altering longwave radiative heating rates. Solar, aerosol, or greenhouse gas perturbations can lead to horizontal variations in atmospheric heating rates and land/ocean contrasts that can alter atmospheric circulations and cloud patterns. These in turn can alter the TOA energy budget. As such, these are not “feedbacks” since they do not involve a response to surface temperature. Changes that occur directly due to the forcing itself, without involving the global-mean temperature, are referred to as “adjustments,” and the corresponding TOA radiative imbalance change is referred to as an “effective” radiative forcing [43]. Because adjustments and feedbacks can occur on similar time scales, separating the two effects poses a major challenge for constraining climate feedback using observations. The clouds will respond to ocean/land and vertical lapse rate adjustments at the same time as they respond to surface temperature change. This will be true regardless of how the forcing is imposed. Clearly, the adjustments will be much stronger immediately following a large instantaneous forcing, but they will also be important over the longer term for a slower continuous radiative forcing.

Because observation-based estimates of climate sensitivity and climate feedback require reliable radiative forcing data [49], uncertainties in aerosol radiative forcing remain a significant source of uncertainty, particularly as observational records become longer. Zelinka et al. [50] show considerable model spread in effective radiative forcing by aerosols among global climate models, and Forster [13] notes that aerosol forcing is the largest contributor to uncertainty in estimates of effective climate sensitivity from long-time scale (multidecadal) observational records (e.g., in situ estimates of ocean heat content change). Quaas [51] highlighted the observational challenges involved in quantifying the contribution by aerosol-cloud interactions, which dominates the uncertainty in climate model radiative forcing. Because aerosol effects can exert significant tropospheric cloud adjustments, diagnosing climate sensitivity from observations is challenging [52].

### Use of Climate Models to Interpret Observations

To improve our understanding of the time dependence of climate feedbacks and their relationship to climate sensitivity, both modeling and observations are essential. However, given the substantial internal variability in the climate system, it is unclear how long an observational record is needed to constrain climate feedback. Chung et al. [52] addressed this question by analyzing climate feedbacks from coupled atmosphere-ocean model simulations using a radiative kernel approach [3, 27, 28]. They computed climate feedbacks from differences between climate states as a function of the length of the time average to define the climate states and the time separation between the climate states. Based upon their results ([52], their Fig. 5f), in order to reduce the upper bound of uncertainty in climate sensitivity by a factor of 2 (equivalent to a 1σ uncertainty in climate feedback of 0.13 W m^−2^ K^−1^), an averaging length of at least 10 years would be required to define the climate states and the climate states would need to be separated by 40 years, thus requiring an observational record of at least 50 years. The main driver for such a long record is from the cloud feedback contribution, which exhibits considerably more variability than other feedback contributions (e.g., lapse rate, water vapor, and surface albedo). Importantly, the Chung et al. [52] analysis does not factor in observational uncertainties, which would further increase the length of the record needed [53].

It is unclear whether or not alternate approaches to the radiative kernel method can be used to help constrain climate feedback with observational records shorter than 50 years. For example, the approach used by Forster and Gregory [8] infers the total climate feedback parameter through a regression of monthly or annual mean TOA radiative flux and surface temperature anomalies. Thus far, the method has been applied to observational records that have been too short to yield robust results (e.g., 15 years or less). It is therefore an open question as to how long a record is necessary. It is also unclear how the data should be averaged, whether monthly, annually, or over a longer period. Forster [13] note that the use of annual averages produces a larger effective climate feedback parameter (smaller effective climate sensitivity, ECS) compared to the use of monthly averages. Estimates based upon longer-term (multidecadal) upper (0–700 m) OHC tendency, radiative forcing, and surface temperature data also point to a larger effective climate feedback (smaller ECS) [13]. Given these discrepancies, there is a need for detailed studies evaluating the strengths and weaknesses of the different methodologies. For example, a model analysis similar to that performed by Chung et al. [52] may help answer some basic questions. In fact, there is a need for more such model simulations to help guide climate observing system requirements in general (these are often referred to as climate Observing System Simulation Experiments (OSSEs)).

Due to the large spread in climate sensitivity among state-of-the-art global climate models, many studies have explored ways of constraining model estimates of climate sensitivity and feedback by evaluating model projections according to how closely present-day observed climate is captured by the models [32, 54–61]. If one or more observed climate variables exhibits a strong relationship between present-day and future climate, then in principle, the observations can be used to identify the models most likely to provide a more accurate estimate of climate sensitivity. While this approach has been remarkably useful for climate model evaluation and diagnosis using historical observations, it has yet to produce a single widely accepted set of observational constraints for narrowing the range in climate sensitivity [57]. A large part of the problem is that since climate models must be relied upon to identify the variables and relationships between present-day and future climate, model weaknesses/deficiencies combined with observational error add considerable uncertainty, limiting our ability to narrow the range in climate sensitivity. Recently, the climate community has adopted the term “emergent constraints” to characterize relationships between intermodel variations in a quantity describing some aspect of observed climate and intermodal variations in a future climate prediction of some quantity [62]. Importantly, in order to qualify as an emergent constraint, the relationships must be physically explainable rather than a possibly fortuitous result.

### Satellite TOA ERB Data Record

**Fig. 1:**
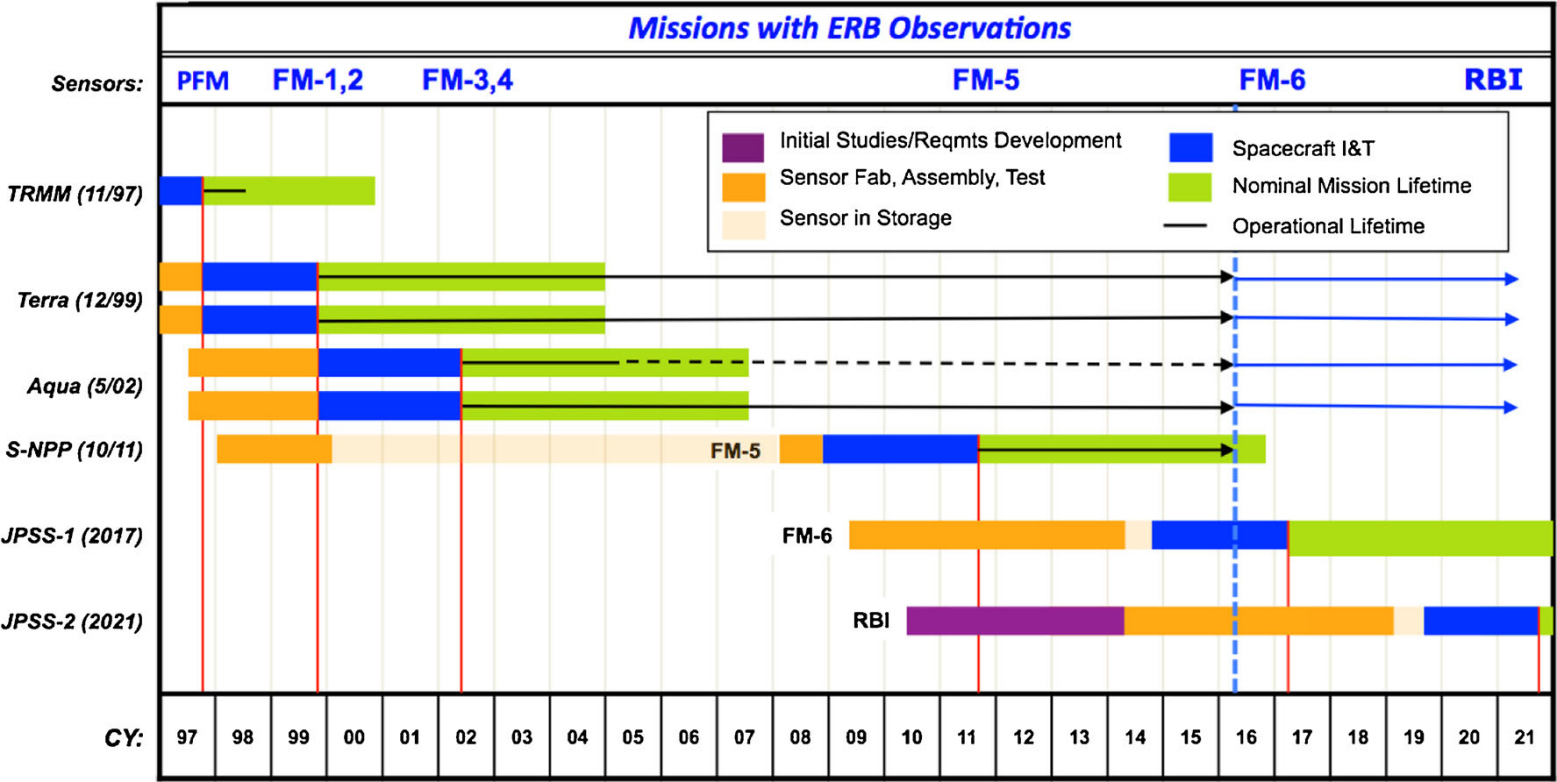
Flight schedule of global ERB monitoring satellite instruments

Given the internal variability of the climate system, improved observational constraints for climate feedback likely require much longer (multidecadal) climate data records. Currently, the longest continuous global ERB observations are from CERES instruments flying on the Terra, Aqua, and S-NPP satellites. A CERES instrument is scheduled for launch on JPSS-1 in 2017, and a follow-on ERB instrument called the Radiation Budget Instrument (RBI) is being built to fly on JPSS-2 in 2021 (Fig. 1).

The reliability of satellite TOA ERB data is influenced primarily by instrument calibration uncertainty, the physical realism of algorithms used to infer geophysical parameters (e.g., clouds, aerosols, and radiative flux), time and space sampling, and the quality of the ancillary input datasets. Which of these factors dominate the error budget is a strong function of the time-space scales that we are interested in resolving. At short time-space scales, the limiting factor is primarily the frequency at which the observations are collected and algorithm uncertainty. At longer time-space scales, radiometric stability of the instruments and long-term consistency of other input data sources matter more. Periodic reprocessing of the entire CERES record is needed to ensure that the data record reflects variations in the climate system as opposed to artifacts associated with algorithm and/or input data changes. Combined use of CERES and imager data enables not only TOA fluxes but also surface radiative fluxes too [63].

**Fig. 2:**
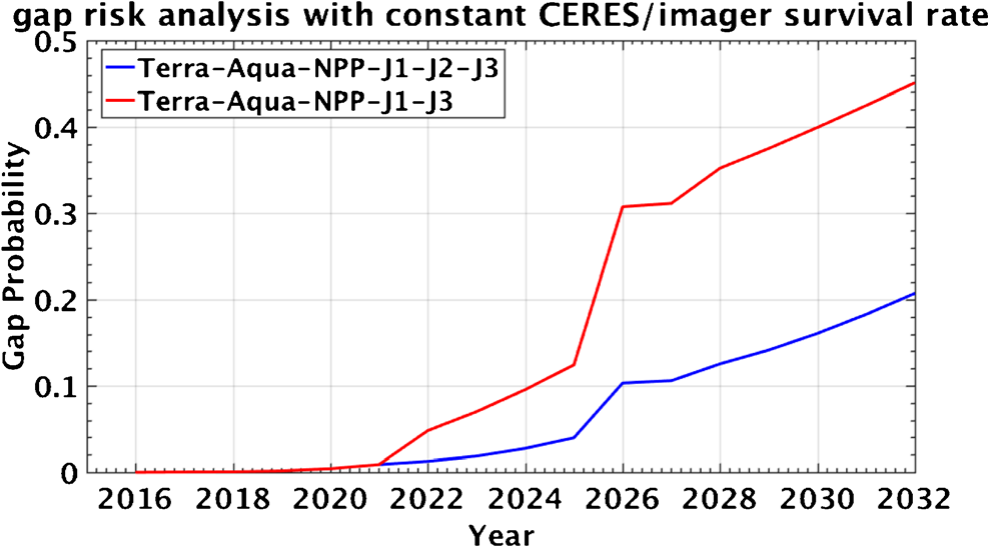
Probability of a data gap in the global satellite ERB time series from present through a given year. The blue curve includes all ERB instruments flying or planned, whereas the red curve excludes the ERB instrument on the J2 satellite

Because the present generation of ERB satellite instruments lacks the absolute calibration accuracy needed to overcome a data gap [64], it is critical that there be at least 1-year overlap between successive satellite missions. There is a high probability that ERB continuity will be achieved through 2030 given the heritage and maturity of current and near-future instruments and data algorithms. Figure 2 provides an estimate of the probability of a gap for the current CERES and RBI flight schedule using historical spacecraft and instrument survival rates [65]. Although not yet official, we assume that a second RBI instrument will fly on the JPSS-3 satellite in 2026. The underlying assumption is that the mission terminates if the primary operational sensor (e.g., MODIS or VIIRS) or spacecraft fails or if fuel becomes too low. The assumed end-of-life dates are 2025, 2021, and 2027 for Terra, Aqua, and NPP, respectively. For the case in which CERES or RBI instruments fly on all available platforms (Terra, Aqua, S-NPP, JPSS-1, JPSS-2, and JPSS-3; blue line), the probability of a gap ranges from 0.15 to 0.20 in the 2028–2030 time frame. The red line shows an alternate scenario, in which RBI does not fly on JPSS-2 but instead flies on JPSS-3. In that case, the gap probability increases markedly to ≈0.45 in 2028 and over 0.50 in 2030.

**Fig. 3:**
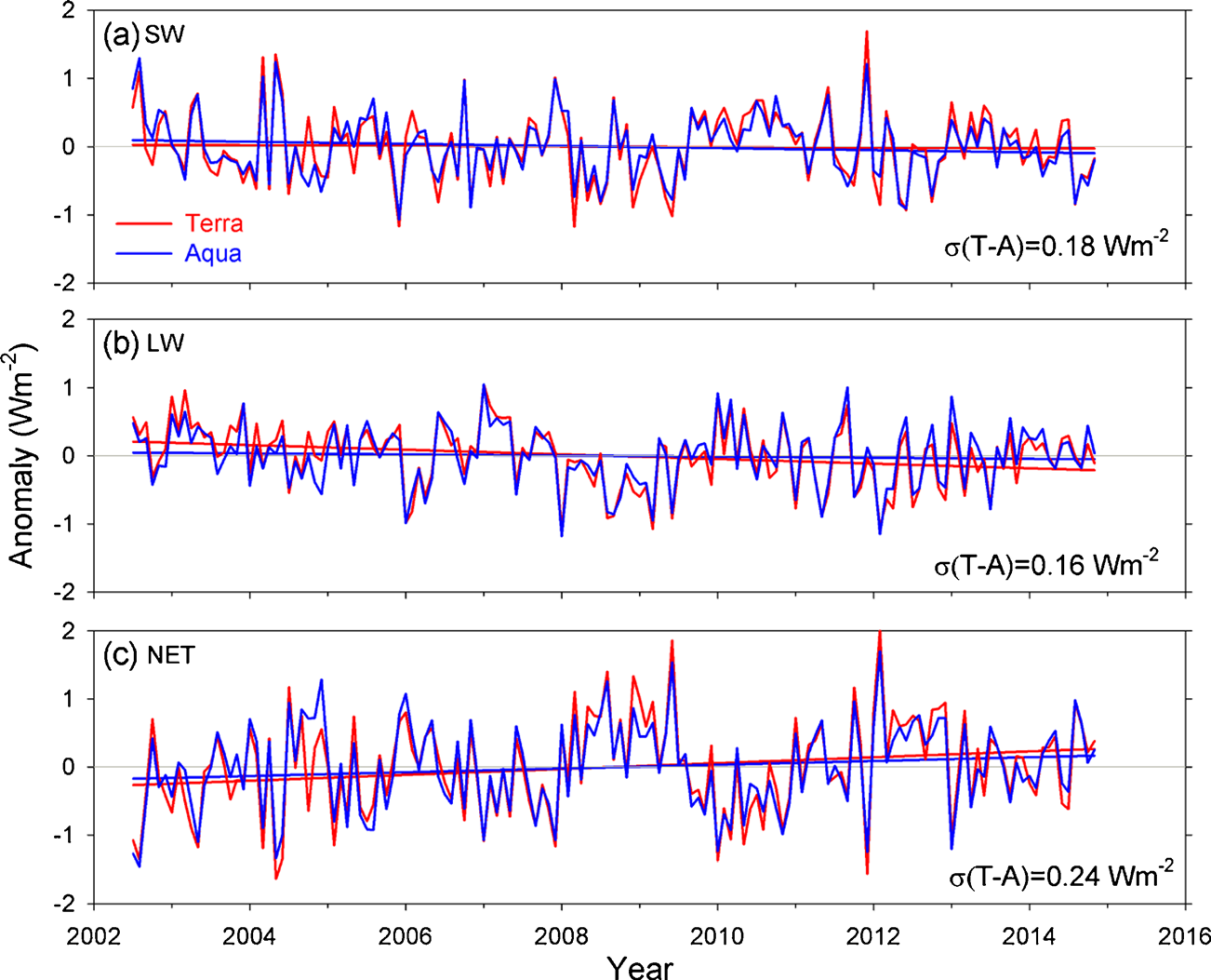
Anomalies in global mean TOA flux for CERES Terra and Aqua from SSF1deg-Edition4A. **a** SW, **b** LW, and **c** net

Thus far, the CERES data products have shown a remarkable ability to track internal variations. To illustrate, Fig. 3a–c shows the TOA flux anomalies from Terra and Aqua using the latest version of CERES data products (SSF1deg-Edition4). The Terra and Aqua trends are within 0.2 Wm^−2^ per decade for SW and 0.3 Wm^−2^ per decade for LW and net at the 95 % significance level. Further improvements are anticipated with the Climate Absolute Radiance and Refractivity Observatory (CLARREO) mission, which will enable intercalibration of many passive satellite instruments in various orbits [66]. A CLARREO Pathfinder mission consisting of a single reflected solar instrument is scheduled to fly on the International Space Station in 2020 as a technology demonstration, which can in principal lead to a more extensive CLARREO mission as proposed in Wielicki et al. [66].

To estimate the uncertainty in the climate feedback parameter due to satellite TOA radiation data using the regression method, Table 1 shows the results using the following three different CERES datasets: Energy Balanced and Filled (EBAF) Ed2.8 [67, 68], Terra SSF1deg-Month Ed4A, and Aqua SSF1deg-Month Ed4A. The SSF1deg-Month Ed4A dataset was just recently released and is currently only available through November 2014. It includes the latest instrument calibration improvements [69], cloud properties, and angular distribution models [70]. We consider the common period of July 2002 to November 2014, when all three datasets are available, and use two surface temperatures, Goddard Institute for Space Studies Surface Temperature Analysis (GISSTEMP) [71] and Hadley Centre Climatic Research Unit version 4 (HadCRUT4) data [72]. Given that the forcing term for such a short period has only a small impact on the climate feedback parameter derived from this method [13], we neglect that term here. The choice of CERES dataset yields an uncertainty in the climate feedback parameter of 0.1 Wm^−2^ K^−1^ (1σ). The uncertainty due to the scatter in the data is approximately 0.5 Wm^−2^ K^−1^ (1σ). Therefore, the uncertainty due to the choice of CERES dataset is a factor of 5 smaller than the noise contribution to the uncertainty. The choice of temperature data also matters. On average, the difference between climate feedback parameters inferred from GISSTEMP and HadCRUT4 is 0.27 Wm^−2^ K^−1^. Using a greater number of temperature datasets, Dessler and Loeb [73] found that the spread in the cloud feedback parameter can be as high as 0.8 Wm^−2^ K^−1^ owing to the choice of the temperature dataset.

**Table 1:** Effective climate feedback parameter and uncertainty (1σ) for July 2002 to November 2014 from regression of CERES net TOA flux and surface temperature anomalies

	GISSTEMP	HadCRUT4
EBAF Ed2.8	−0.89 ± 0.56	−1.12 ± 0.63
SSF1deg Ed4A (Terra)	−0.74 ± 0.64	−1.10 ± 0.75
SSF1deg Ed4A (Aqua)	−0.68 ± 0.61	−0.90 ± 0.69
Average	−0.77 ± 0.60	−1.04 ± 0.69
Standard deviation	0.11	0.12

The effective climate feedback parameter also exhibits a surprisingly strong sensitivity to the period considered. Using the EBAF2.8 dataset, an effective climate feedback parameter was determined for 2001–2013 and 2001–2015 (Table 2). For the 2001–2015 period, the effective climate feedback parameter decreased by a factor of 3 to 5 for monthly averages and 5 to 14 for annual averages compared to that for 2001–2013. This is likely associated with the inclusion of the strong El Niño in 2014–2015. Consistent with Forster [13], the effective climate feedback parameter tends to be larger using annual averages.

**Table 2:** Effective climate feedback parameter for 2001–2013 and 2001–2015 using monthly and annual averages in the regression

Date range	Monthly averages		Annual averages	
	GISSTEMP	HadCRUT4	GISSTEMP	HadCRUT4
2001–2013	−1.13 ± 0.52	−1.18 ± 0.58	−3.6 ± 1.6	−4.5 ± 1.8
2001–2015	−0.35 ± 0.43	−0.27 ± 0.47	−0.48 ± 1.1	−0.32 ± 1.1

TOA radiation data were from the CERES EBAF Ed2.8

In order to evaluate the influence of a data gap on satellite-derived effective climate feedback parameter, we consider monthly anomalies between 2001 and 2015 for CERES EBAF Ed2.8 global net TOA flux and GISSTEMP surface air temperature (Table 3). With no data gap, the effective feedback parameter is −0.35 ± 0.43 Wm^−2^ K^−1^. Assuming a 1-year gap in 2008 and no calibration change following the gap, the effective feedback parameter becomes −0.19 ± 0.45 Wm^−2^ K^−1^. However, when calibration error is introduced by imposing a ±2 % discontinuity to the period following the gap, the effective feedback parameter changes further by ±0.05 Wm^−2^ K^−1^. Thus, in this example, introducing a gap of 1 year has a substantial impact on the effective feedback parameter derivation both due to the data that is missing and to a lesser extent the calibration difference between the period prior to and after the gap.

**Table 3:** Effective climate feedback parameter using monthly averages in the regression

Data range	Calibration change following gap	Effective feedback parameter (Wm^−2^ K^−1^)
2001–2015	None	−0.35 ± 0.43
2001–2015 (gap in 2008)	None	−0.19 ± 0.45
2001–2015 (gap in 2008)	+2 %	−0.25 ± 0.46
2001–2015 (gap in 2008)	−2 %	−0.14 ± 0.46

Net TOA radiation data are from the CERES EBAF Ed2.8, and surface air temperature anomalies are from GISSTEMP

## Conclusions

The large spread in equilibrium climate sensitivity (1.5–4.5 °C) has not narrowed appreciably during the past 30 years, owing primarily to uncertainties in the representation of climate feedbacks in climate models. While observationally based estimates suggest that climate feedbacks collectively enhance the temperature response to a forcing, the magnitudes of the climate feedback parameter estimates vary greatly. Use of observations to constrain climate models and narrow the range in climate sensitivity has thus been largely unsuccessful so far. Even with perfect observations, there are limitations to what can be achieved with short observational records because different forcings produce different patterns of warming, and feedbacks respond differently to different spatial variations in temperature. At multidecadal time scales, coupled atmosphere-ocean simulations show considerable time variation in the effective global climate feedback parameter, providing a further imperative for continuing to collect stable, long-term climate observations. At the same time, there is a need to continue to revisit the underlying assumptions used in the traditional forcing-feedback framework, with an emphasis on how climate models and observations can best be utilized to reduce uncertainties not only in climate sensitivity but also the spatial and temporal patterns of climate change. The climate models can also provide important insights on the observational requirements needed to make progress in this area. For example, dedicated climate OSSEs can help establish the suite of climate variables that need to be observed over multiple decades, at what accuracy, temporal/spatial resolution, etc., and can also help guide how the data should most effectively be analyzed. The climate OSSE framework is also critical to help guide process-based observational requirements (both satellite and field campaigns) in model development efforts. Ultimately, improved representation of climate feedbacks in models requires realistic, physically based parameterizations. Process observations play a critical role in model development, while longer-term observations are needed to assess model representation of climate variability and change at interannual and decadal time scales.

On the observational side, it is more critical than ever to commit to sustained long-term and stable measurements of key variables used to estimate climate feedbacks. These include solar irradiance, TOA Earth radiation budget, in situ ocean heat storage, aerosols, clouds, ice sheet and sea ice volume, and temperature/humidity profiles. For passive remote sensing satellite measurements, overlap between successive missions is needed to avoid data gaps in the record and to ensure a consistent calibration throughout. There is also a need to fly dedicated radiance calibration missions (e.g., CLARREO) that can help improve the accuracy and stability of a wide range of passive sensors (including weather satellite instruments) in various orbits, thereby making our observing system more accurate. This represents a paradigm shift from the typical satellite mission that targets observations of specific geophysical variables. However, given that the climate system changes and feedback we are trying to observe are small compared to the internal variability of the climate system, and given that at long time scales instrument calibration is the dominant error source, such a paradigm shift is critically needed.
